# How do clinicians use implementation tools to apply breast cancer screening guidelines to practice?

**DOI:** 10.1186/s13012-018-0765-2

**Published:** 2018-06-07

**Authors:** Heather Armson, Stefanie Roder, Tom Elmslie, Sobia Khan, Sharon E. Straus

**Affiliations:** 1 Department of Family Medicine, University of Calgary, Calgary, AB Canada; 2 The Foundation for Medical Practice Education, McMaster University, Hamilton, ON Canada; 3 Departments of Family Medicine, and Community Medicine and Epidemiology, University of Ottawa, Ottawa, ON Canada; 4 Knowledge Translation Program, Li Ka Shing Knowledge Institute, St. Michael’s Hospital, Toronto, ON Canada; 5 Department of Medicine, University of Toronto, Toronto, ON Canada

**Keywords:** Clinical practice guidelines, Guideline implementation, Implementation tools, Implementation strategy, Breast cancer screening

## Abstract

**Background:**

Implementation tools (iTools) may enhance uptake of guidelines. However, little evidence exists on their use by primary care clinicians. This study explored which iTools clinicians used and how often; how satisfied clinicians were with the tools; whether tool use was associated with practice changes; and identified mediators for practice change(s) related to breast cancer screening (BCS).

**Methods:**

Canadian primary care providers who are members of the Practice-Based Small Group Learning Program (*n* = 1464) were invited to participate in this mixed methods study. An educational module was discussed in a small group learning context, and data collection included an on-line survey, practice reflection tools (PRTs), and interviews. The module included both the Canadian Task Force on Preventive Health Care revised guideline on BCS and iTools for clinician and/or patient use. After discussing the module and at 3 months, participants completed PRTs identifying their planned practice change(s) and documenting implementation outcome(s). Use of the iTools was explored via online survey and individual interviews.

**Results:**

Seventy participants agreed to participate. Of these, 48 participated in the online survey, 43 completed PRTs and 14 were interviewed. Most survey participants (77%) reported using at least one of seven tools available for implementing BCS guideline. Of these (78%) reported using more than one tool. Almost all participants used tools for clinicians (92%) and 62% also used tools for patients. As more tools were used, more practice changes were reported on the survey and PRTs. Interviews provided additional findings. Once information from an iTool was internalized, there was no further need for the tool. Participants did not use tools (23%) due to disagreements with the BCS guideline, patients’ expectations, and/or experiences with diagnosis of breast cancer.

**Conclusion:**

This study found that clinicians use tools to implement practice changes related to BCS guideline. Tools developed for clinicians were used to understand and consolidate the recommendations before tools to be used with patients were employed to promote decision-making. Mediating factors that impacted tool use confirmed previous research. Finally, use of some iTools decreased over time because information was internalized.

**Electronic supplementary material:**

The online version of this article (10.1186/s13012-018-0765-2) contains supplementary material, which is available to authorized users.

## Background

An interval of approximately 17 years has been suggested as the gap between publication of research findings and application to patient care [1, 2]. One approach to shorten this interval has been the widespread development and distribution of clinical practice guidelines (CPG). CPGs, however, have not made a significant impact on uptake of recommendations into clinical practice [3–8]. Only 55% of patients receive the recommended health care they need [6, 9]. While there are many explanations for the lack of implementation [10], the development of tools to facilitate application of guideline recommendations has been proposed by guideline developers as one approach to appropriately address this gap between knowledge and implementation [3, 11]. A knowledge translation (KT) tool is defined as “an intervention in a form of a tangible product or resource that can be used to implement best evidence into practice” [12]. One classification of KT tools identifies three categories: implementation tools (iTools) such as printed educational materials and decision aids that facilitate practice change; resource planning tools that support implementation processes; and evaluation tools such as quality indicators [13]. This paper focuses on iTools, which can be directed at clinicians and/or patients to clarify goals of care, understand evidence, assess the risks and benefits of treatment options, and enable informed decision-making [14, 15]. Interviews with healthcare professionals support the need for tools to implement guideline recommendations [16].

Systematic reviews suggest that iTools such as guideline summaries, algorithms, point-of-care checklists, and reminders enhance compliance with guideline recommendations [17–19]. However, a systematic review evaluating the effectiveness of iTools developed and disseminated with guidelines identified only four studies [20]. The tools utilized in these studies targeted only healthcare professionals; none targeted healthcare organizations or patients. Tools included educational workshops tailored to barriers, paper-based educational materials, order forms, or reminders. Only two of the four studies assessed adherence to guidelines, reporting a median 13.5% greater adherence in the intervention group [20].

The features of iTools have been explored by a number of researchers. MacDermid et al. (2013) [12] described a taxonomy for KT tools based on the Knowledge-to-Action cycle with tools directed at each stage of the model. Gagliardi et al. (2014) [3] identified the features of ideal guideline iTools (e.g., stated tool objectives, identification of target users, provision of instructions on tool use, evidence cited for tool content) and proposed a framework for evaluating and adapting existing tools, or developing new tools. However, only 8.7% of guidelines reviewed had accompanying iTools and many did not possess most of the features identified by the framework [3].

Despite the identification of the importance of tools to the implementation of guidelines and the development of checklists and frameworks for assessing these tools, very little research has examined the use of iTools in clinical practice [21]. This study focused on this aspect of iTool use based on the breast cancer screening (BCS) guideline of the Canadian Task Force for Preventative Health (CTFPHC) [22]. A collaboration was created between CTFPHC [23] and the Foundation for Medical Practice Education (FMPE) [24]. The mandate of the CTFPHC is to create and disseminate guidelines designed to optimize the quality of preventive care delivered in primary care [25]. FMPE serves as a knowledge user through the development and dissemination of evidence-based educational modules that provide tools (practice aids, algorithms, recommendations, chart aids, and/or resources) intended to facilitate practice implementation and practice change by clinicians.

In Canadian women, breast cancer is the most common form of cancer and the second leading cause of cancer death [26, 27]. Screening has been shown to be of benefit in certain age groups. Screening rates in Canada, however, suggest that only 54% of eligible women access screening as recommended [28]. The CTFPHC guideline made a number of recommendations that balance the benefits and harms of BCS for women who have no pre-existing risk factors [22]. These included confirming the previous recommendation against breast self-exam and supporting mammography screening of women 50–69 years. However, timing of mammography was changed from yearly to every 2–3 years. Previous recommendations not to screen women 40–49 years with no pre-existing risk factors were also confirmed. A new recommendation was that mammography screening occurs in the 70–74-year-old age group. Another change was the recommendation to discontinue screening breast exams by clinicians [22]. All of the recommendations made by the CTFPHC were considered weak recommendations based on low to moderate quality evidence and therefore, the revised guidelines emphasized the need for discussions between clinicians and patients exploring the balance between harms and benefits and patients’ values and preferences. To facilitate implementation of these recommendations into practice, specific tools (Table 1) were developed or adapted for use by clinicians and patients.

**Table 1:** Implementation tools included in the module and with the guidelines [22, 32]

Implementation tools	Location of tool	Target user	Type of tool	Description—information provided in tool
Screening Recommendations for breast cancer with mammography	Module	Clinician	Printed educational \material—Table	Screening recommendations with mammography: ➢ For women 40–49 years –(2012) “recommendation against routine screening” ➢ For women 50–74 years –(2012) “routine screening every 2–3 years” (70–74 years was new addition) Includes: numbers needed to screen to save one life from breast cancer; harms, and adverse outcomes Clinical consideration for implementation: provide information to patients, use decision aid, and use electronic health records to flag screening reminders for patients 50–74 years
Screening recommendations for clinical breast exams and breast self-exams	Module	Clinician	Printed educational material—Table	Screening recommendations for clinical breast exam and breast self-exam: ➢ Clinical breast exam—changed from previous (2001) “every year for women 50–69 years” to current (2012) “recommend against” ➢ Breast self-exam—changed from previous (2001) “not recommended” was strengthened to current (2012) “recommend against” Clinical considerations for implementation: clinical breast exams still appropriate if there are concerns about abnormal breasts
Discussion video: CTFPHC breast cancer video	Hyperlink to CTFPHC website	Clinician	Video	Video (12 min) directed at clinicians, exploring strategies for patient discussion around breast cancer screening issues
Patient handout: breast cancer screening—what is the right choice for me?*	Module	By patient	Decision aid	Questions/answers addressing breast cancer screening: ➢ What is breast cancer screening? ➢ Should I have a breast exam? ➢ Who should have a mammogram? Includes the following: Patient check list to review each benefit and harm as it relates to them specifically Pictorial representation of benefits and harms of breast cancer screening for women age 40–49 years.
Patient handout: CTFPHC patient algorithm	Hyperlink to CTFPHC website	By/with patient	Printed educational material—algorithm	Algorithm guides individuals re mammography: ➢ For women 40–49 years—“suggest not screening with mammography” ➢ For women 50–74 years—“suggest scheduling mammogram every 2–3 years” ➢ For women 75+ − “suggest discussing the benefits and harms of mammography with a family physician”
Patient handout: CTFPHC benefits and risks	Hyperlink to CTFPHC website	With Patient	Decision aid	Patient handout describing benefits and the risks of breast cancer for women between 40 and 49, 50–69, and 70–74 years of age. It includes a pictorial representation of outcome of screening in each age group including false positives, biopsy, and mastectomy
Patient handout: CTFPHC FAQs for patients	Hyperlink to CTFPHC website	Patient	Printed educational material—FAQ for patients	Highlights that CTFPHC recommends that women aged 50–74 schedule a mammogram every 2–3 years. Questions/answers address: • High risk; meaning of screening; best way to screen; • Benefits and harms associated with mammography; • Reasons for different recommendations for different age groups; • Recommendations for breast self-examination and clinical breast exams.

*CTFPHC* Canadian Task Force on Preventive Health Care, *FAQ* frequently asked questions

*Based on Public Health Agency Canada and CTFPHC handouts

This study explored (a) which iTools clinicians reported using and how often, (b) how satisfied clinicians were with the tools used, (c) perceived usefulness of tools, (d) whether tool use was associated with reported practice changes, and (e) mediators (barriers and enablers) for reported practice change(s) related to BCS.

## Methods

**Fig. 1:**
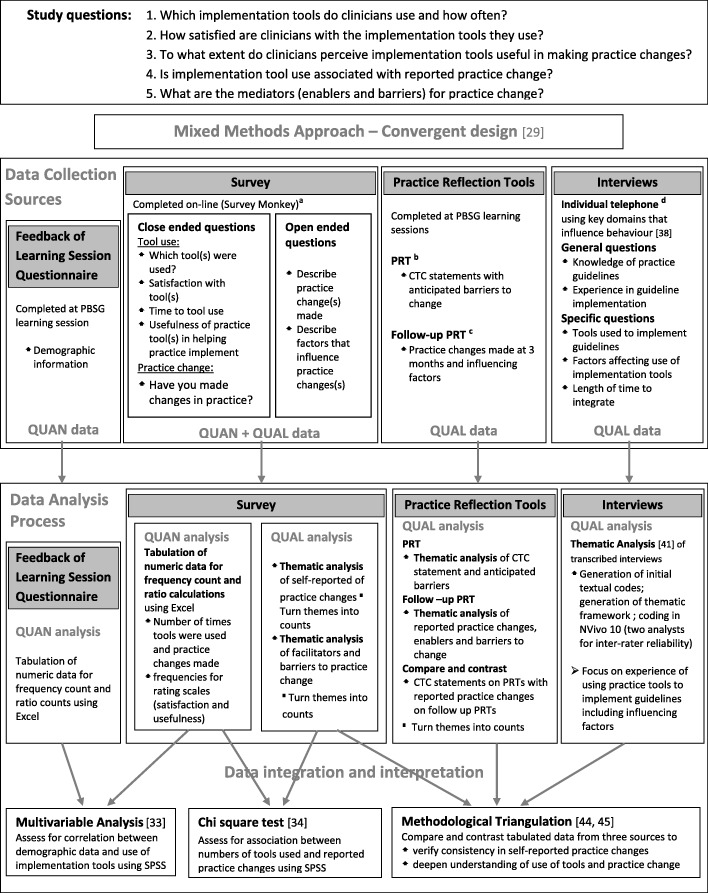
Overview of mixed methodology approach to study how clinicians use implementation tools. CTC = commitment to change; PBSG = practice-based small group; PRT = practice reflection tool; QUAL = qualitative; QUAN = quantitative; a-see Additional file 1; b-see Additional file 2; c-see Additional file 3; d-see Additional file 4

A convergent mixed method approach [29] explored the use of iTools in this study (see Fig. 1 for study overview). Quantitative data was collected from a questionnaire and survey. Open-ended survey questions, practice reflection tools, and interviews provided qualitative data.

### Recruitment

FMPE operates the Practice-Based Small Group (PBSG) learning program for practicing clinicians (family physicians and nurse practitioners). Small groups of clinicians meet within their local communities across Canada once a month to discuss case-based educational modules. Trained peer facilitators moderate these sessions providing an interactive educational platform for continuing professional development [30].

**Fig. 2:**
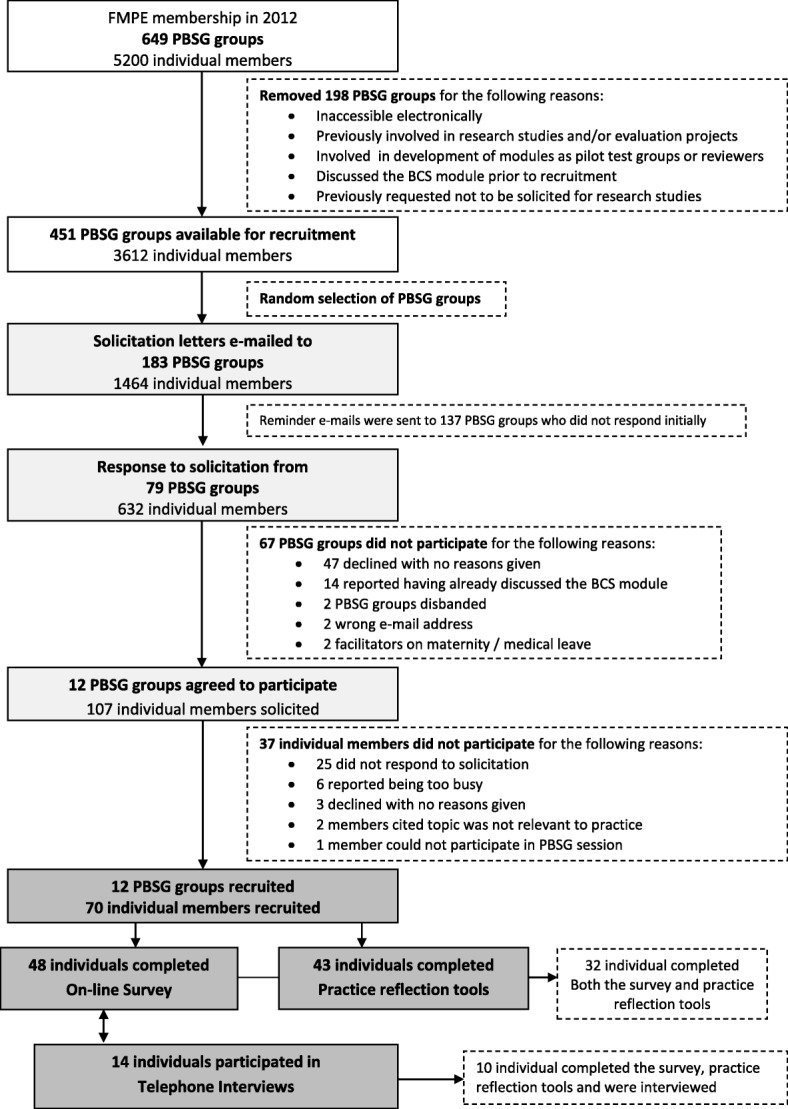
Flowchart showing the solicitation and recruitment efforts for the study. FMPE = The Foundation for Medical Practice Education; PBSG = practice-based small groups; BCS module = Breast Cancer Screening module

Between September 2012 and January 2013, 183 randomly selected PBSG facilitators (from 451 PBSG groups) were solicited via e-mail to participate in the study (Fig. 2). Approximately 3 weeks after initial solicitation, reminders were sent to facilitators who did not respond. Once a group (6–12 members) agreed to participate in the study, individual members confirmed participation by signing a consent form.

### Study protocol

The CTFPHC revised their BCS guideline in November 2011 based on a need to target average-risk women in a manner that would balance effective screening with harm reduction [31]. In May 2012, FMPE developed and distributed to their membership an educational module on *Breast Cancer Screening: Conversations with Women* (BCS module), based on the revised CTFPHC guideline to assist in interpreting the evidence and facilitate conversation with patients about the benefits and risks of screening [32]. iTools were developed and incorporated into both the CTFPHC guideline and the BCS module.

Study participants discussed the BCS module between September 2012 and March 2013 during a regularly scheduled PBSG session. Participants were asked to complete a PRT, a normal part of their meetings, and a paper-based questionnaire specific to this study. A PBSG discussion about the success of proposed practice changes (from the PRT) occurred approximately 3 months after the initial meeting. This session included the completion of a follow-up PRT as usual. Outside the regular meetings, participants were asked to complete an online survey. All survey participants were invited to take part in one-on-one telephone interviews.

### Implementation tools

Seven iTools were available with the BCS module (Table 1): three as part of the module and four as web links within the module. Three tools were targeted to primary care clinicians and four tools directed to patients. iTools were created for the updated BCS guideline using established methods including user-centered design and usability testing by the target audience [25].

All tools were compared to the ideal features outlined by Gagliardi et al. 2014 [3]. CTFPHC tools had all features of an ideal iTool while FMPE tools missed two features (description of methods used to develop and evaluate the tools).

### Data collection and analysis

There were four data sources (Fig. 1): Feedback of Learning Session questionnaire; online survey; practice reflection tools; and telephone interviews. Each participant was assigned an ID number that was applied to all study documents to de-identify data. Chronology of the data collection is shown in Table 2. Data analysis was both quantitative and qualitative and is described below for each data source.

**Table 2:** Timeline of publications, recruitment of study participants and four sources of data collection

Timeline	Publications					
November 2011	CMAJ-CTFPHC revised Breast Cancer Screening Guidelines					
Timeline	Recruitment of study participants	Data collection				
		Feedback of learning session questionnaire^a^	Practice reflection tools		Online survey^c^	Telephone interviews^d^
			PRT^a^	Follow-up PRT^b^		
September 2012	x	x	x			
October 2012	x	x	x			
November 2012	x	x	x			
December 2012	x	x	x	x	x	
January 2013		x	x	x	x	
February 2013		x	x	x	x	
March 2013		x	x	x	x	
April 2013				x	x	
May 2013				x	x	x
June 2013				x	x	x
July 2013						x
August 2013						x

*CMAJ* Canadian Medical Association Journal, *CTFPHC* Canadian Task Force for Preventative Health, *FMPE* The Foundation for Medical Practice Education, *PBSG* practice-based small group, *PRT* practice reflection tool

^x^Time frame of study activity (recruitment and data collection)

^a^Completed by participants during initial PBSG learning session

^b^Completed by participants during a follow-up PBSG review session approximately 3 months later

^c^Participants completed on-line survey on their own time

^d^Individual telephone interviews were scheduled at times convenient for the participant

#### Feedback of learning session questionnaire

The paper-based questionnaire had demographic questions (gender, location of practice, type of practitioner, practice experience, and patient population served) and provided an opportunity to give feedback on the session.

Data was tabulated in Microsoft Excel 2007 for frequency counts and ratio calculations.

#### Online survey

Three months (December 2012–June 2013) after the initial PBSG session, participants received an e-mail with a link to an online survey (Survey Monkey Inc.; Additional file 1). The survey explored the different tools participants used in clinical practice and any factors that affect tool use and practice change. The survey featured 12 itemized questions and consisted of close- and open-ended questions. Close-ended questions on tool use and satisfaction were either dichotomous, multiple choice, or on a 7-point Likert rating scale (e.g., *completely dissatisfied* to *completely satisfied)*. Open-ended questions were used to provide information on successful practice changes, on mediators to practice changes, and reasons for not making any practice change(s). E-mail reminders were sent 1 week after the follow-up PBSG review session. Additional reminders were sent at 1-week intervals to a maximum of four.

Open-ended survey responses were extracted and grouped into statements for self-reported practice changes and statements for mediators to practice changes. Each group of statements was organized into common themes (SR), these were reviewed and discussed by team members (SR, HA, TE). Coding schemes for each group of statements were created and then used to code all the open-ended responses (SR).

All survey data were tabulated in Microsoft Excel 2007 for frequency counts and ratio calculations. This included counts for iTools use, satisfaction ratings, usefulness of the tools, the number of practice changes made, and counts of themes.

To determine the extent of any relationships across the quantitative and qualitative data, variables of interest were pulled into cross-tab tables for multivariable analysis [33]. To assess for an association between the number of tools (0, 1, 2, 3, > 3) used and self-reported practice changes (no change, already changed, made change), a chi-square test was performed [34]. Significance level was set at *p* < 0.05. All statistical analyses were done using IBM SPSS Statistics for Windows version 23.0 [35].

#### Practice reflection tools

A PRT was completed at the conclusion of the initial PBSG session. The PRT (Additional file 2) facilitates reflection on clinical practice, helps to identify practice gaps, and encourages documentation of planned practice changes in the form of enhanced commitment-to-change statements [36]. Three months after the initial discussion, participants received a copy of their PRT and were asked to complete a follow-up PRT (Additional file 3) summarizing efforts to change practice including encountered mediators to change.

Commitment-to-change statements on the PRT were matched with “changes made” statements on the follow-up PRT. Themes related to practice changes and mediators to practice changes were identified and tabulated in Microsoft Excel 2007 for frequency counts (SR). This information was reviewed by team members (HA, TE) and subsequently cross-referenced with the survey data to verify the consistency of self-reported practice changes and identification of mediators to practice change (SR).

#### One-on-one telephone interviews

All survey respondents were invited to participate in 1-h, semi-structured individual telephone interviews between April and June 2013. The interview guide (Additional file 4) was based on the theoretical domains framework [37–39], which “identifies influences on health professional behavior related to implementation of evidence-based recommendation” [40]. The audio-taped interviews conducted by one of two experienced interviewers (SK, SR) explored the mediating factors behind implementing change(s) in clinical practice, with a specific focus on the factors influencing the use of BCS iTools. The interviews were transcribed and de-identified.

Two research team members (AM, SR) independently coded the first three interviews using a thematic analysis approach [41]. An initial list of themes was developed and discussed with the whole research team. Themes were organized into a coding scheme. This scheme was used to code the remaining interviews using NVivo™ 10 software program [42] (AM, SR). New themes were added to the coding scheme as necessary. Any discrepancies were discussed and resolved. The transcripts and the coding were reviewed by a third team member (HA) to confirm analysis. Data saturation was determined after completion of thematic analysis and was defined as the point at which no new themes were identified with additional interviews [43].

The interview data was compared to the survey and PRT data to deepen understanding of the use of tools and impact on practice change. Themes from each data source were summarized and then compared [44, 45].

## Results

### Participants

Of the 183 PBSG groups that were solicited, 79 PBSG groups responded. Twelve groups and 70 of 107 individual members in those groups (65%) consented to participate in the study (Fig. 2).

Participants were from six Canadian provinces: five groups from Ontario, two from each of British Columbia and Nova Scotia, and one from each of Manitoba, New Brunswick, and Saskatchewan. Family physicians comprised 91% (64/70) and nurse practitioners 9% (6/70) of the participants. More than 60% of participants were involved in group practices (43/70), and there was a predominance of female participants (80%, 56/70). There was a fairly equal distribution in years of practice: 1–10, 11–20, and 21–30 years (20, 24, and 20%, respectively); of the remaining participants, 13% were in practice for more than 30 years; 23% did not answer the question. Sixty percent of participants practiced primarily in an urban/suburban setting.

### Completion rates for each data source

Of the 70 study participants, 48 (69%) completed the online survey; 43 (61%) completed both the PRT and follow-up PRT and 14 (20%) participated in 1-h individual telephone interviews (Fig. 2).

Twenty-one of the 48 survey participants responded to invitations to be interviewed. Of these, seven participants declined, citing lack of time or lack of information additional to their survey responses. Fourteen - 1hr telephone interviews were completed between May and August 2013. These interview participants included 10 who had implemented practice changes and four who did not make their proposed changes. Fourteen interviews were sufficient to reach data saturation as all themes were identified within the first 11 interviews.

### Data utilized to address each of the study questions

#### Which iTools clinicians reported using and how often?

Survey data indicated that 77% (37/48) of survey participants reported using at least one tool. Of these 37 participants, 8 used one tool, 13 used two tools, and 16 used three or more tools (Table 3).

**Table 3:** Tool use as reported by survey participants

Tool use	% of total survey participants	Tools for clinicians only	Tools for patients only	Tools for clinicians and patients
No tool	23% (11/48*)	–	–	–
Tools used	77% (37/48)	14/37 (38%)	3/37 (8%)	20/37 (54%)
Number of tools used by 37 participants	% of survey participants using tools	Clinician tools	Patient tools	Clinician and patient tools
One tool	22% (8/37**)	7	1	–
Two tools	35% (13/37)	7	2	4
Three tools	29% (11/37)	–	–	11
Four tools	3% (1/37)	–	–	1
Five tools	8% (3/37)	–	–	3
Seven tools	3% (1/37)	–	–	1

*Total number of survey participants

**Total number of survey participants using tools

**Fig. 3:**
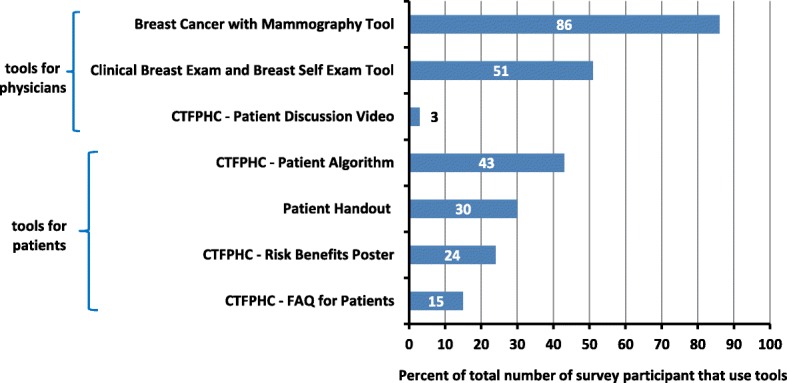
Summary of implementation tool used. Percent of survey participants (*n* = 37) who used the various breast cancer screening tools provided or referenced in the Breast Cancer Screening module. The description of each tool can be found in Table 1. Note: numbers do not total 100% as more than one tool could be selected

Tools for clinicians were used by nearly all participants (92%; 34/37), particularly the BCS with Mammography and CBE/BSE tools (Fig. 3). Tools for patients were used by nearly two-thirds of survey participants (62%; 23/37), the most popular being the Patient Algorithm (Fig. 3). Only one survey participant watched the patient discussion video.

A combination of iTools for clinicians and patients were selected by more than half of survey participants (Table 3). When only one tool was chosen, tools for clinicians were preferred by 88% (7/8) of survey participants (Table 3). When two tools were used, tools for clinicians were still the dominant choice, but there was also an increased likelihood that a tool for patients would also be used. When three tools were used, a combination of tools for clinicians and patients were used. Few survey participants used more than three tools (Table 3).

Although the PRTs did not specifically ask about iTool use, 44% (19/43) of the participants who completed the PRTs mentioned iTools. Under the “most useful information” section on the PRT (11/19) references were made to the tools provided within the BCS module, e.g. “concise charts with screening recommendations for breast cancer with mammography and patient handout for breast cancer screening” (100–788). Participants also made reference to tools under the PRT section on enablers (4/19) e.g. “visual documentation to inform patients of advantages and disadvantages of mammography” (140–657). Participants (2/19) also referred to tools on the follow-up PRT e.g., “used handout material to discuss with patients especially in 40-50-year age group” (80–344).

Interview participants mentioned the tools they found contributed to effective conversations with women about BCS:

"I used the patient handout from the module, ... the FAQ for patients, I have made some copies of that. I do not have them sitting out but I’ll bring them out if the discussion warrants it, … I like algorithms in general … I think they are a nice graphic, a lot of information that you can take in quickly and easily, so in general I am an algorithm fan." Int #12.

Tools to be used with patients were used more often when conversation became more challenging:

“…probably have not used it [patient handout] that many times. It just more depends on if the patient... most patients accept it fairly easy and so we talk about it, have a discussion and that’s it. I don’t use the tool, but if they are a little bit more in lines of, my friend had this or, how come I’m not getting this done? Then I will use it… say, half a dozen times maybe” Int #13.

Interview participants explained that once information had been internalized, there was no further need to use the iTools:

“And then [screening for CBE and BSE tool], I used that a lot at the beginning… just to remind myself … there’s evidence to show that we won’t reduce a woman’s risk of death from cancer … with the CBE, and also the self -breast exam- I won’t reduce her chance of dying and I could cause harm…now that I’ve internalised it I haven’t really needed to create a link…” Int #8.

No association between demographic data and frequency of tool use could be ascertained as the numbers were too small to perform a multivariable analysis.

#### How satisfied were clinicians with the tools used?

On average 70% of survey participants reporting tool use rated their use as mostly or completely satisfactory (Additional file 5–Table A); less than half (47%; 9/19) of participants were mostly or completely satisfied with the CBE/BSE tool.

Although not a PRT question, one participant recorded on their PRT: “I liked the numbers/statistics showing how many cancers vs. false positives vs. biopsies would be found / done for each of the age groups if we screen” (20–399).

Interview participants perceived the tools as informative, with clear and concise information that helped facilitate discussions with patients (Table 4). However, some interview participants felt that some of the tools were not at the appropriate comprehension level for a lay person (e.g., benefits and risk poster). A further suggestion was that patient handouts such as the FAQ for patients should be limited to one page.

**Table 4:** Interview participants’ perception of implementation tools

Implementation tool	Participants’ perception
TOOLS to be used by CLINICIANS	
Screening recommendations for breast cancer with mammography - in PBSG module	Majority found tool very informative Some felt they could internalize information, therefore not requiring continued access to tool
Screening recommendations for clinical breast exams and breast self-exams - in PBSG module	General consensus that tool simple and straightforward. Some found tool simple enough for use in educating medical students and residents Primary barrier—disagreed with CBE recommendations
Discussion video: CTFPHC breast cancer video - on CTFPHC website	Only one user for this tool Many had difficulty accessing, opening or downloading the tool Others preferred reading information to watching a video
TOOLS to be used with or by patients	
Patient handout: breast cancer screening—what is the right choice for me?—in PBSG module	Tool useful for facilitating discussions with patients, especially the provision of a visual estimation of risk Primary barrier for implementation—availability of tool and time constraints for discussing the tool
Patient handout: patient algorithm—hyperlink to CTFPHC website	Total consensus on value of algorithms, information is concise and clear Could easily be internalized No noted barriers
Patient handout: benefits and risks poster—hyperlink to CTFPHC website	Tool viewed as a valuable teaching tool for patients Good information with supporting statistics Several participants felt that concepts may be beyond the comprehension level of the average layperson
Patient handout: FAQs for patients - hyperlink to CTFPHC website	A valuable tool. Good information sheet to hand out to patients that have questions. Tool aesthetically pleasing and therefore an incentive for patient to read. Limited awareness and restricted accessibility were barriers to implementation. Two pages may be too lengthy for patients (tool developers consider reducing to one page for all age groups?)

*CBE* clinical breast exam, *CTFPHC* Canadian Task Force for Preventive Health, *FAQ* frequently asked questions*, PBSG* practice-based small group

#### To what extent do clinicians perceive iTools as useful?

Survey participants agreed and strongly agreed with the statement that iTools are useful in making practice changes 70% of the time (Additional file 5–Table B).

When considering practice change, some participants (4/19) documented on their PRT that they will “increase informing [patients to] use handouts provided” (30–106) and reported on the follow-up PRT that the handout material was used (2/19).

Interview participants reported that the tools were useful for both answering their questions about screening and educating patients:

“I think initially for me the appendices, both of them [BCS with Mammography and the CBS/BSE tools], for going over the new recommendations, they were helpful. And again, it was more for me to solidify in my mind ... the what and why of the changes.” Int #9.

The combination of the evidence in the module and the related tools were identified as increasing the effectiveness of conversations and facilitating discussion with women about BCS:

“I always found that [BCS] a difficult area to discuss … this module gave me some evidence and some tools that made that conversation with that particular group of women…breast cancer is one of the things women are really concerned about, and it’s always in the public eye….so it kind of raises a lot of anxieties in the exam room. So, it’s nice to see a conversation aide and different ways of answering questions.” Int #11.

#### Is iTool use associated with reported practice change?

Seventy-seven percent (37/48) of survey participants reported making one or more practice change(s) related to BCS. Specifically, they did less mammography screening in those patients who were under 50 or over 75 years of age (65%; 24/37), conducted fewer CBE (38%; 14/37), and had more discussions with patients about guideline recommendations (32%; 12/37). All of the participants who made practice changes indicated making changes within 2 weeks following the PBSG session. Practice changes were still being applied three or more months after the initial discussion of the BCS module.

When no practice change was made (23%; 11/48), survey participants stated that they either perceived themselves as already following the BCS recommendations (*n* = 5) or were not convinced that a change was necessary (*n* = 6).

**Fig. 4:**
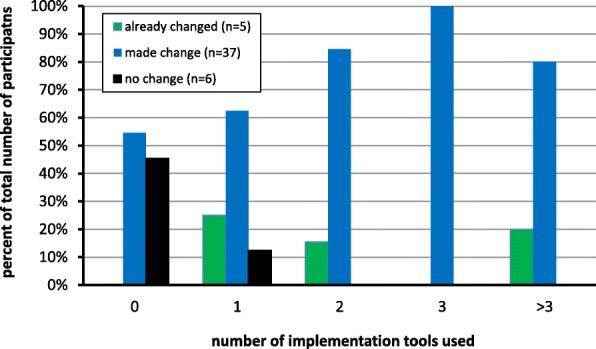
Relationship between tool use and self-reported practice change. Data is shown for those that reported having made a change prior to study (already changed), made at least one practice change (made change), and those that did not make a change (no change)

Survey data were used to assess for association between number of tools used and reported practice change. Although the graph shown in Fig. 4 suggests that there is a relationship between tool use and self-reported practice change (i.e., survey participants using more than one tool were more likely to report making a practice change), the numbers were too small to claim statistical significance using chi-square test (contingency tables for chi-square testing showed that 60% of expected values were less than 5) [34]. All participants who stated that they did not make a change as they were already following the guideline reported having used one or more iTools. Most of the participants (5/6) who were not convinced to change reported not using tools.

Data from the PRTs also provided specific information on practice changes related to BCS. Seventy-nine percent of (34/43) participants followed through on at least one of their planned practice changes such as reducing the number of both mammogram referrals (56%, 19/34) and breast exams conducted in asymptomatic women < 50 years (44%, 15/34) and increasing patient discussions (47%; 16/34). Eighteen percent (6/34) mentioned using tools (i.e., patient handouts) to educate patients about the harms/benefits of screening.

Data from the survey and the PRTs related to self-reported practice changes were compared to determine level of consistency between the data collection methods. Almost all (96%; 27/28) of the participants who reported making practice change(s) on the survey also indicated on their follow-up PRT that they made *some or all* of their planned practice changes and the majority (82%; 23/28) reported using iTools to do so. The majority (80%; 4/5) of survey participants who reported making no practice change also stated on their PRTs that they had not intended to make a change.

#### What are mediators for reported practice change(s) related to BCS?

Mediators for practice change were explored across three data sources—survey, PRTs, and interviews. Statements from each data source were categorized as barriers or enablers (see Additional file 6 for sample statements). Depending on the perspective of survey participants, level of evidence for the guideline was reported either as an enabler (confidence in the evidence) or a barrier (disagreement with the evidence) to practice change. The most common barrier to following the guideline was concerns that the recommendations would not benefit patients.

Participants who reported following through on the practice changes provided one or more statements on the enablers (*n* = 28) and barriers (*n* = 20) on the follow-up PRT. Reported enablers to practice changes were the evidence-based information in the guideline (9/28) and in the BCS module (10/28), discussions with peers in the learning sessions (6/28), the application of iTools (3/28), and exchanges with patients about the guideline recommendations (3/34). Reported barriers to practice change were lack of evidence about benefit of the recommendations for patients (3/20), conflicting information with different provincial guidelines (4/20), patients’ expectations of being screened for breast cancer (6/20), fear on the part of both patients and clinicians of missing a diagnosis (4/20), and problems associated with having patient handouts readily accessible (3/20).

Interview participants identified a number of factors that influenced their decision to use a tool and to follow the BCS guideline. Many of the mediating factors were similar to those identified in the survey responses including the perceived conflict with other guidelines, the role of a previous negative experience, and patient expectations. In addition, a finding only seen in the PRT and interview data was that clinicians reported difficulties in changing a previous routine activity. The role of PBSG discussions was a theme identified as both an enabler and barrier to implementation across all data sources. Additional themes only identified from the interview data related to the specific aspects of the use of tools, such as accessibility, practice application, time constraints, and patient literacy (Table 5).

**Table 5:** Aspects of implementation tools that influence the decision for use

Descriptor	Enablers	Barriers
Accessibility	Integrating into EMR so access occurs within patient charts. Accessibility through FMPE and CTFPHC websites.	Tools not compatible with some EMRs or required approval to be input, and therefore not usable. Paper may be difficult to find. “…. it’s very hard to pull out the paper in the middle of a discussion because I can never find it, and when I actually do need to find something it is quite frustrating when I cannot find the paper that I am looking for.” Int #10
Application	Supplement to patient consultations and increased patient education.	Some clinicians may use tools as substitute for actual discussions with patients. If not part of routine practice; unlikely to use. Not suitable for simple messages.
Number of tools		Space constraints for retaining/displaying so many tools.
Time constraints		Time consuming to explain tools to patients, to integrate into EMRs and to use consistently.
Patient literacy		Literacy level may be too high. Tools not accessible for either visual or hearing impaired.

*CTFPHC* Canadian Task Force for Preventive Health, *EMR* electronic medical record, *FMPE* The Foundation for Medical Practice Education

## Discussion

The use of iTools in routine clinical practice has previously received little attention. This study explored the use of iTools on uptake of BCS recommendations provided by the CTFPHC guideline [22].

### Implementation tool use

Seven iTools created for the updated BCS guideline were directed at both new recommendations and reiterations of recommendations from previous guidelines. Seventy-seven percent of survey participants reported using at least one of the seven iTools, with the majority (78%) using more than one tool. Tools which confirmed previous guideline recommendations were still used, specifically, supporting mammography screening in the 50–69-year age group and discouraging screening in the 40–49-year age group (86%). Even participants who were already following these recommendations reported using at least one of the iTools.

Tools chosen were dependent on participants’ acceptance of the BCS recommendations. Participants voiced their disagreements with the BCS guideline recommending discontinuation of CBE and reported lower use of the CBE/BSE tool compared to the screening for mammography tool. This finding was not surprising, as previous studies have shown that tools are not used when there are disagreements with guidelines [46]. Even when the recommendation around discontinuing CBE was adopted, participants struggled with potentially missing a cancer diagnosis and patients’ expectations of the previously routine CBE. This de-implementation challenge has been described in previous studies [47, 48].

### Satisfaction with iTools

Tool users were generally satisfied with the BCS iTools. Inclusion of desirable features of tools [3] may have contributed to the satisfaction, however, no evidence exists that clinicians consider these features when choosing tools. Participants described the use of tools for patients as helpful in the decision-making process. They used these tools in direct conversations with patients rather than as handouts to be reviewed at home. Specific aspects of the tools were described as enhancing use: simplicity, pictorial representations of risk, patient centered. Participants reported liking algorithms.

Easy access to iTools in paper format in the BCS module appeared to enhance use. However, except for the videotape, many participants also used the CTFPHC tools which were hyperlinked within the module suggesting that differences in access location may not be crucial.

### Perceived usefulness of iTools

The use of iTools for clinicians was described in the interviews as facilitating clinician understanding and comfort with the material contained in the guideline *prior* to initiating discussions with patients. The subsequent use of iTools for patients was dependent on a clear understanding of the risks and benefits associated with screening. The sequential use of tools, i.e., the use of tools for clinicians first followed by tools for patients, has not been previously reported in the literature and should be explored further.

Adherence to tool use [8, 49] was noted by survey participants up to 3 months after initial implementation. However, in a few instances, participants reported initially using tools as a reminder of the guideline recommendations but felt the tools were no longer necessary once information had been internalized. This was reported with both tools for clinicians and patients—an important finding suggesting tool impact would be significantly underestimated if use alone were measured. Further work is required to understand this internalization process and whether ongoing practice continues to reflect guideline recommendations once the tool is no longer used.

### iTools and reported practice change(s)

Most survey participants who reported using tools indicated making practice changes related to BCS, including less mammography screening, fewer CBE in low-risk patients, and more discussions with patients about the benefits and harms of BCS.

There appeared to be a relationship between the number of tools used and reports of practice changes but the sample size did not allow statistical assessment of this preliminary observation. These findings around practice change and tool use provide some support for the purported usefulness of guideline implementation tools [3, 16, 50]. However, this relationship, based on self-reports, needs to be followed up with more objective data from practice audits.

### Mediators for reported practice change(s) related to BCS

Mediating factors related to tool use for implementing guideline recommendations have been reported in the literature, and many of these factors were seen in this study including awareness, accessibility, level of clinician and patient agreement, evidence for recommendations, patient literacy, outcome expectancy, and consistency of recommendations across different guidelines [6, 46, 51–55]. Disagreement with guideline recommendations was one major mediating factor presented in this study as discussed above. While interview participants identified the lack of robust evidence as a reason for not implementing some of the guideline recommendations, none articulated the important role of decision support tools in facilitating an informed discussion with patients about harms and benefits especially in the context of a weak recommendation. In the BCS guideline, all of the recommendations were weak yet physicians either accepted the recommendations or dismissed them which usually dictated whether information would be presented to patients. Further education of clinicians may facilitate more effective use of decision aids.

Another factor is the ease with which tools can be trialed without the need for additional resources [56]. This could explain the negligible use of the clinician video on patient education. Accessing the video required an internet link and 12 min to view. Interview participants identified this as time-consuming and not fitting with their workflow as opposed to the straightforward use of the other internet or paper-based tools.

### Limitations

This study looked at a specific guideline based on one clinical topic. There may be generalizability to other screening guidelines given the similar content and context, but application to non-screening guidelines would require further investigation. The sample size was small in terms of the quantitative components of the study, whereas the participant numbers for the qualitative data collection was acceptable. Recruitment was challenging at least partially because of publication of the CTFPHC recommendations and the release of the BCS module before study recruitment. There was a higher female to male ratio (80% female; 20% male) relative to their proportion within the PBSG program (65% female; 35% male) likely due to the topic under study. Clinician gender has been identified as a factor in mammography screening with male physicians having a two-fold reduction in screening compared to their female counterparts [57]. Female physicians have also been shown to provide more preventative services than their male counterparts [58–61], especially in breast screening [62]. The increased proportion of female clinicians participating in this study may compromise the generalizability of the findings to male clinicians.

Finally, although three different data collection methods were used in this study (survey, PRTs, interviews), all were dependent on self-report. The comparison across data sets increases the trustworthiness of the findings. Previous work on the accuracy of self-report related to practice change at least partially mitigates this potential risk [63–66]. However, correlation with practice audits would be helpful in confirming the findings of this study in the future.

## Conclusion

The study provides new information about the use of iTools in clinical practice. Use of specific tools was reported by most clinicians, and many used more than one tool supporting the recommendation to increase tool development to support implementation of the CPG recommendations [3, 11, 16]. An exploration using objective measures of tool use would bolster these findings.

The reported sequencing of tool use suggests clinicians may need to consolidate their understanding and application of the guideline recommendations in their clinical practice context prior to the use of iTools directed at patients. Further confirmation and clarification of this finding may impact future tool development.

A surprising finding was that clinicians did not consider the quality of the evidence when deciding about the use of patient decision aids, particularly in the context of recommendations with weak evidence. This suggests that clinician education may be required about their use.

The relationship between iTool use and reported practice change is an intriguing finding and requires further exploration with a larger sample and objective measures of practice change.

Finally, researchers need to be aware that actual use of iTools may decrease over time because information has been internalized.

## Additional files

Additional file 1: Online survey questions. Study participants completed on on-line survey (Survey Monkey Inc.) 3 months after a practice-based small group learning session on breast cancer screening. The aim of the survey was to explore the different tools that participants used in clinical practice and determine any factors that affect the use of tools and practice change. (DOCX 15 kb)

Additional file 2: Practice reflection tool. Study participants completed a paper-based practice reflection tool (PRT) at the conclusion of their initial practice-based small group learning session on breast cancer screening. The PRT facilitates individual reflection on clinical practice, helps identify practice gaps, and encourages documentation of planned practice change(s) in the form of enhance commitment-to-change statements. (DOCX 81 kb)

Additional file 3: Follow-up practice reflection tool. Study participants completed a paper-based follow-up practice reflection tool (follow-up PRT) 3 months after their initial practice-based small group learning session on breast cancer screening. The follow-up PRT facilitates individual reflection on previous planned practice change(s) and encourages documentation of the outcome of the planned practice change(s), including enablers and barriers to making practice change(s). (DOCX 75 kb)

Additional file 4: Interview guide. Individual 1-h semi-structured telephone interviews were conducted with consenting study participants by one of two experienced interviewers to explore the mediating factors behind implementing change(s) in clinical practice, with a specific focus on the factors influencing the use of breast cancer screening implementation tools. (DOCX 18 kb)

Additional file 5: Satisfaction and usefulness of implementation tools. Tables provide the results of the ratings given by tool users for satisfaction with implementation tools (Table A) and the tools’ usefulness in implementing practice changes (Table B) with respect to the breast cancer screening guidelines. (DOCX 18 kb)

Additional file 6: Perceived enablers and barriers to practice change. Table provides the themes for perceived enablers and barriers to reported practice changes from three data collection sources: surveys, practice reflection tools, and interviews. (DOCX 20 kb)

## Abbreviations

BC: British Columbia
BCS: Breast Cancer Screening
BSE: Breast self-exam
CBE: Clinical breast exams
CMAJ: Canadian Medical Association Journal
CPG: Clinical practice guideline
CTC: Commitment-to-change
CTFPHC: Canadian Task Force for Preventative Health
dx: Diagnosis
EMR: Electronic medical record
FAQ: Frequently asked questions
FMPE: The Foundation for Medical Practice Education
Int: Interviewee
iTool(s): Implementation tool(s)
KT: Knowledge translation
NS: Nova Scotia
OBSP: Ontario Breast Screening Program
PBSG: Practice-based small group
PRT: Practice reflection tool
pts.: Patients
QUAL: Qualitative
QUAN: Quantitative
REB: Research Ethics Board
SPSS: Statistical Package for Social Sciences
