# Supplementary material for: How do clinicians use implementation tools to apply breast cancer screening guidelines to practice?
Source: Implement Sci. 2018 Jun 7;13:79. doi: 10.1186/s13012-018-0765-2 (PMC5992659; doi:10.1186/s13012-018-0765-2)
Supplement: Supplementary file 1 — Online survey questions. Study participants completed on on-line survey (Survey Monkey Inc.) 3 months after a practice-based small group learning session on breast cancer screening. The aim of the survey was to explore the different tools that participants used in clinical practice and determine any factors that affect the use of tools and practice change. (DOCX 15 kb) [file 13012_2018_765_MOESM1_ESM.docx]

**Additional file 1**– On-line survey questions

1. Since the PBSG meeting, have you made changes in your practice regarding breast cancer screening? Yes / No
2. Please describe the practice change(s) you made with respect to breast cancer screening (open ended)
3. Please describe any factors the facilitated the practice change(s) you made with respect to breast cancer screening (open ended)
4. Please describe any factors that hindered the practice change(s) you made with respect to breast cancer screening (open ended)
5. Please describe the reason(s) why you did not make any practice change with respect to breast cancer screening (open ended)
6. Please indicate how long after the PBSG meeting you began to implement the described change(s) to your breast cancer screening practice.

Up to 1 week after the PBSG session

1-2 weeks after the PBSG session

3-4 weeks after the PBSG session

Over 4 weeks after the PBSG session

7. Are you still implementing the described breast cancer screening change(s)? Yes / No

If no, please explain why you have stopped implementing the described practice change(s). (open ended)

8. In the past 3 months with which of the following age groups did you discuss the risks and benefits of breast cancer screening? (check all that apply)

Patients <49 years

Patients 50-74 years of age

Patients >75 years

The next set of questions were asked for each of the seven practice tools provided in or referenced to in the Breast Cancer Screening Module (see Table 1). A visual of each practice tool was provided. The names of the practice tools were: 1. The screening recommendations for Breast Cancer with Mammography; 2. The screening recommendations for Clinical Breast Exam and Breast Self Exam; 3. Patient Handout; 4. CTFPHC Breast Cancer Screening Discussion Video; 5. CTFPHC Breast Cancer Screening Patient Algorithm; 6. CTFPHC Risk vs. Benefits Poster; 7. CTFPHC on Preventive Health Care Breast Cancer Screening FAQ for patients.

1. Have you used “*Name of practice tool*” (tool is shown below)? Yes / No

If no, please explain why you did not use this tool. (open ended)

1. Please describe your satisfaction with the screening recommendations for “*Name of practice tool*” 7 point Likert scale (Completely dissatisfied to completely satisfied; not applicable)
2. “*Name of practice tool*” was useful in helping implement my described practice changes 7 point Likert scale (strongly disagree to strongly agree; not applicable)

Last question of the survey asked the following:

1. Please, list any other continuing professional development activities related to Breast Cancer Screening, other than the PBSG sessions that you have engaged in over the past 3 months
