# Supplementary material for: How do clinicians use implementation tools to apply breast cancer screening guidelines to practice?
Source: Implement Sci. 2018 Jun 7;13:79. doi: 10.1186/s13012-018-0765-2 (PMC5992659; doi:10.1186/s13012-018-0765-2)
Supplement: Supplementary file 2 — Practice reflection tool. Study participants completed a paper-based practice reflection tool (PRT) at the conclusion of their initial practice-based small group learning session on breast cancer screening. The PRT facilitates individual reflection on clinical practice, helps identify practice gaps, and encourages documentation of planned practice change(s) in the form of enhance commitment-to-change statements. (DOCX 81 kb) [file 13012_2018_765_MOESM2_ESM.docx]

**Additional file 2**– Practice Reflection Tool
