# Supplementary material for: How do clinicians use implementation tools to apply breast cancer screening guidelines to practice?
Source: Implement Sci. 2018 Jun 7;13:79. doi: 10.1186/s13012-018-0765-2 (PMC5992659; doi:10.1186/s13012-018-0765-2)
Supplement: Supplementary file 3 — Follow-up practice reflection tool. Study participants completed a paper-based follow-up practice reflection tool (follow-up PRT) 3 months after their initial practice-based small group learning session on breast cancer screening. The follow-up PRT facilitates individual reflection on previous planned practice change(s) and encourages documentation of the outcome of the planned practice change(s), including enablers and barriers to making practice change(s). (DOCX 75 kb) [file 13012_2018_765_MOESM3_ESM.docx]

**Additional file 3**– Follow- up Practice Reflection Tool
