# Supplementary material for: How do clinicians use implementation tools to apply breast cancer screening guidelines to practice?
Source: Implement Sci. 2018 Jun 7;13:79. doi: 10.1186/s13012-018-0765-2 (PMC5992659; doi:10.1186/s13012-018-0765-2)
Supplement: Supplementary file 4 — Interview guide. Individual 1-h semi-structured telephone interviews were conducted with consenting study participants by one of two experienced interviewers to explore the mediating factors behind implementing change(s) in clinical practice, with a specific focus on the factors influencing the use of breast cancer screening implementation tools. (DOCX 18 kb) [file 13012_2018_765_MOESM4_ESM.docx]

**Additional file 4**

**Interview Guide**

**Project Title:** Sustaining behaviour change: A knowledge translation partnership between the Canadian Task Force on Preventive Health Care and the Foundation for Medical Practice Education

**Preparation for interview:**

Prior to the interview:

- E-mail copies / images of breast cancer screening tools to study participant to have on hand during the interview
- E-mail copy of Breast Cancer Screening module to study participant to have on hand during interview
- Identify timeframe when Breast Cancer Screening module was reviewed by group
- Have a copy of the personal PRT and survey results as reference during the interview

**THE INTERVIEW**

***Introduction:***

Hello Dr __________,

My name is xxx and I work at the Foundation as a research coordinator since 2007. My colleague, yyy, a research coordinator with the Knowledge Translation group at St. Michael’s Hospital in Toronto, and I are interviewing a number of PBSG members who have discussed the 2012 Breast Cancer Screening Module.

***Purpose of Interview:***

We thank you very much for agreeing to participate in this study. Today we would like to discuss with you the breast cancer screening guideline on which the module was based and the tools provided to you to help you implement the guideline into your practice. The purpose of this interview is to explore barriers and/or facilitators to implementing change(s) in clinical practice, with a specific focus on what factors influence the use of breast cancer screening tools. Any information you provide use during this interview will help guide the refinement of the breast cancer screening tools.

***Permission to tape the interview session:***

We would like to tape this session to ensure accuracy of information you will give us today. To ensure confidentiality, when the interview is transcribed we will remove your name and any other names of individuals that may be mentioned. Once the conversation today has been transcribed the recording will be destroyed.

Do you have any questions? [Pause]

Do, I have your permission to proceed? [Pause] □ Confirmation by participant

We have sent you a copy of the breast cancer screening module and the breast cancer screening tools to help orient our discussion today. Do you have them available?

- Initiate recording

***Interview begins:***

I realize it may have been some time since you and your PBSG group discussed the Breast Cancer Screening module, but perhaps you could start by telling us……...

1. What have been the key messages related to the new breast cancer screening guideline produced by the Canadian Task Force on Preventive Health Care? [knowledge]
2. Have you visited the Canadian Task Force website for the breast cancer screening guidelines? How useful was this in your understanding of the guidelines? [knowledge]
3. How important do you think was Breast Cancer Screening module and /or the small group discussion to your understanding of the breast cancer screening guideline? [knowledge]

Next we would like to ask you about …

1. What was your experience of implementing breast cancer screening recommendations in your clinical practice as outlined in the guideline? [skills/believes about capabilities/optimism]
2. Did the small group discussion provide any suggestions to enhance implementation of this guideline? [skills/believes about capabilities/optimism] If yes, please explain.
3. During your small group session, did you discuss with your colleagues any specific practice changes you would like to make with respect to the breast cancer screening guideline? If yes, what were they? [skills/believes about capabilities/optimism]

Next we have a few specific questions about the breast cancer screening tools that you may or may not have used…

1. Please, describe the breast cancer screening tools you have used to implement practice changes. [skills/believes about capabilities/optimism]

[PROMPT: look at breast cancer screening tools, explain some were provided in module appendix, others were provided through web links]

If tools have been used continue with next question

If tools have not been used skip to question 10 (Please, describe factors that affect your ability to use or not use the breast cancer screening tool [environmental context and resources])

Yes, tools have been used … otherwise skip

1. Why did you choose this tool / these tools? [name the tool] [skills/believes about capabilities/optimism]
2. How often do you use this tool / these tools? [skills/believes about capabilities/optimism]

If tools have been used or not used continue here …

1. Please describe any factors that may affect your ability to use or not use the breast cancer screening tools. [environmental context and resources]
2. Did the small group discussion have any influence in your decision to use or not use this tool/ these tools? [skills/believes about capabilities/optimism]
3. How well do you think the breast cancer screening tools were / could be integrated into your clinical practice? [role and identity]
   1. How long does it take to integrate this change/ these changes into you clinical practice? [nature of behaviours]
4. What are your thoughts on the positive and negative consequences of using the breast cancer screening tools in your clinical practice? [beliefs about consequences]
5. Please identify any system(s) that are in place to ensure that the changes made are sustained over the long term. [Prompt: Have you added any information into patient health records or EMR? What type of information/tools did you add / flag?] [nature of behaviours]
6. Do you think the ongoing PBSG sessions help sustain practice change (s) over time? [nature of behaviours]
7. How do others influence your breast cancer screening practice and use of the guideline tools (e.g. patients, colleagues, other professionals)? [social influence]
8. Have you discussed the guidelines or tools with other physicians beside your PBSG group? [nature of behaviours] If yes, please describe whether you perceive a difference in practice regarding using the screening guidelines tools between physicians in your PBSG group and other physicians outside of your PBSG group*.* [nature of behaviours]

We still have some time left for a couple of general questions:

1. Please, comment on your view of the credibility of the Canadian Task Force for Preventive Health Care in developing the breast cancer screening guidelines for physicians.
2. Please, comment whether the breast cancer screening guideline conflicts with any other guidelines that you can think of.

***End of Interview:***

We are at the end of the interview. Do you have any additional or final comments you would like to make?

Thank the interview for participating.

- Stop recording
