# Supplementary material for: How do clinicians use implementation tools to apply breast cancer screening guidelines to practice?
Source: Implement Sci. 2018 Jun 7;13:79. doi: 10.1186/s13012-018-0765-2 (PMC5992659; doi:10.1186/s13012-018-0765-2)
Supplement: Supplementary file 5 — Satisfaction and usefulness of implementation tools. Tables provide the results of the ratings given by tool users for satisfaction with implementation tools (Table A) and the tools’ usefulness in implementing practice changes (Table B) with respect to the breast cancer screening guidelines. (DOCX 18 kb) [file 13012_2018_765_MOESM5_ESM.docx]

| **Table A – Rating of SATISFACTION with implementation tools** | | | | | | | | | | |
| --- | --- | --- | --- | --- | --- | --- | --- | --- | --- | --- |
| **KT Tools** | ***Completely dissatisfied*** | ***Mostly dissatisfied*** | ***Somewhat dissatisfied*** | ***Neither satisfied or dissatisfied*** | ***Somewhat satisfied*** | ***Mostly satisfied*** | ***Completely satisfied*** | | **Total** | |
| Screening Recommendations for Breast Cancer with Mammography | 0 | 0 | 1 | 2 | 5 | 20 | 4 | | **32** | |
| Screening Recommendations for Clinical Breast Exams and Breast Self Exams | 0 | 0 | 2 | 3 | 5 | 5 | 4 | | **19** | |
| Discussion Video: CTFPHC Breast Cancer Video | 0 | 0 | 0 | 0 | 0 | 1 | 0 | | **1** | |
| Patient Handout: Breast Cancer Screening – What is the Right Choice for Me? | 0 | 0 | 0 | 0 | 3 | 4 | 4 | | **11** | |
| Patient Handout: CTFPHC Patient Algorithm | 0 | 0 | 0 | 1 | 1 | 11 | 3 | | **16** | |
| Patient Handout: CTFPHC Benefits and Risks Poster | 0 | 0 | 0 | 1 | 2 | 5 | 1 | | **9** | |
| Patient Handout: CTFPHC FAQs for Patients | 0 | 0 | 0 | 2 | 0 | 1 | 2 | | **5** | |
| **Total** | **0** | **0** | **3** | **9** | **16** | **47** | | **18** | | **93** |

**Additional file 5 -** Ratings given by **TOOL USERS** for satisfaction with implementation tools (Table A) and the tools’ usefulness in implementing practice changes (Table B) with respect the breast cancer screening guidelines

| **Table B – Rating of USEFULNESS of tools in implementation of practice changes** | | | | | | | | | | |
| --- | --- | --- | --- | --- | --- | --- | --- | --- | --- | --- |
| **KT Tools** | ***Strongly disagree*** | ***disagree*** | ***Somewhat disagree*** | ***Neither agree or disagree*** | ***Somewhat agree*** | ***Agree*** | ***Strongly agree*** | | **Total** | |
| Screening Recommendations for Breast Cancer with Mammography | 0 | 0 | 0 | 2 | 6 | 17 | 7 | | **32** | |
| Screening Recommendations for Clinical Breast Exams and Breast Self Exams | 0 | 0 | 1 | 1 | 4 | 9 | 4 | | **19** | |
| Discussion Video: CTFPHC Breast Cancer Video | 0 | 0 | 0 | 0 | 0 | 1 | 0 | | **1** | |
| Patient Handout: Breast Cancer Screening – What is the Right Choice for Me? | 0 | 0 | 0 | 2 | 3 | 2 | 4 | | **11** | |
| Patient Handout: CTFPHC Patient Algorithm | 0 | 0 | 0 | 2 | 1 | 8 | 5 | | **16** | |
| Patient Handout: CTFPHC Benefits and Risks Poster | 0 | 0 | 0 | 1 | 3 | 2 | 3 | | **9** | |
| Patient Handout: CTFPHC FAQs for Patients | 0 | 0 | 0 | 1 | 1 | 0 | 3 | | **5** | |
| **Total** | **0** | **0** | **1** | **9** | **18** | **39** | | **26** | | **93** |

*CTFPHC= Canadian Task Force on Preventive Health Care*
