# Supplementary material for: How do clinicians use implementation tools to apply breast cancer screening guidelines to practice?
Source: Implement Sci. 2018 Jun 7;13:79. doi: 10.1186/s13012-018-0765-2 (PMC5992659; doi:10.1186/s13012-018-0765-2)
Supplement: Supplementary file 6 — Perceived enablers and barriers to practice change. Table provides the themes for perceived enablers and barriers to reported practice changes from three data collection sources: surveys, practice reflection tools, and interviews. (DOCX 20 kb) [file 13012_2018_765_MOESM6_ESM.docx]

**Additional file 6 -** Perceived enablers and barriers to reported practice changes from three data collection sources

| ***Themes*** | ***Data collection sources*** | | | |
| --- | --- | --- | --- | --- |
|  | ***Survey data*** | | ***Practice Reflection Tool data*** | ***Interview data*** |
| **Enablers** | | | | |
| **Practice tools for patient consultations** | *“evidence based recommendations to share with patients to understand the change in opinion” (130-289)* | | *“visual documentation to inform patients of advantages and disadvantages of mammography” (140-657)* | “*we all liked that one at the back with the dots and the representing each woman, and then showing just the one woman would not die from breast cancer. So we all try to keep that around our desk, the patient handout.”* Int#2 |
| **Information in the Breast Cancer Screening module** | *“directly facilitated by the information in the PBSG module” (130—283)* | | *“Information from the module” (30-103)*  *“ The breast cancer screening module*  *and discussion with my colleagues” (130-284)* | “*I did find the information very useful, especially, like I say, because I needed to make that adjustment to my practice with the CBE, … it was important for me to read it and digest it … anything that ‘s Cole’s notes version in a busy office is always good”* Int#9 |
| **Evidence outlining the benefits/risks of screening** | *“reviewing the evidence (lack of evidence) for this screening manoeuvre” (120-212)*  *“Reviewing the strength of evidence regarding low risk screening” (50-988)* | | *“evidence around the lack of benefit with CBE” (30-109)* | *“..evidence against performing clinical breast examination routinely, and then the evidence that does not support offering mammography before age 50 in women who are not high risk…”* Int#11  *“So had there really been good, strong evidence, if you could have shown me all the harm that physicians cause by picking up benign lumps and having them biopsied and all this kind of stuff, and all the things we do nasty, then I think I could have easily changed like I did with self breast examination”* Int #3 |
| **PBSG group discussion** | *“reviewing the evidence in a different forum (I had previously reviewed the new guidelines, but this was PBSG) and discussing evidence based practices with group members” (20-399)* | | *“discussion with my colleagues” (20-390)*  *“group discussion” (130-283)* | *“I find the small group very helpful because we sit, we have a lot of discussion, we’re coming from two different clinics, similar populations but a lot of experience at the table, so it’s definitely helpful to hear their opinions and how to put it into work, you know, put it into your practice.”* Int#2 |
| **Patient awareness, media information** | *“media information about guidelines and that US also moved to routine screening after 50 helps as more consistent messages given to patients” (80-344)* | | *“patients actually accepted this with little explanation” (140-653)*  *“Publicity re: changes patients saw in media” (80-349)* | *“breast cancer is one of the things women are really concerned about, and it’s always in the public eye, and always been, you know, advertising by marketing stuff for it, so it seems to be right-there-right-now in your face”* Int #2 |
| **BARRIERS** | | | | |
| **Unconvinced that the breast cancer screening guidelines would benefit patients / lack of evidence** | *“my lack of personal support for the change in guidelines” (10-784)*  *“issue of no CBE and BSE: understand the reasoning, but asking patients to know their own body and for clinicians to get used to doing exams seem still a good thing rather than detrimental. How do we graduate a new class of physicians who don’t do any of these exams? Also, my own personal bias plays factor as I detected my own breast cancer on BSE.” (80-344)*  *“the fact that breast cancers in this age group are usually more aggressive than in the older groups” (90-141)* | *“ I have a hard time accepting that the risk of screening in this group outweighs the benefits” (100-787)*  *“I feel this is a minimal invasive exam that could potentially benefit a patient without considerable risk” (30-103)*  *“not convinced that guidelines would benefit patients” (40-438)*  *“not enough evidence to make me change my current practice of screening for breast cancer” (100-790* | | *“It’s not the tool so much itself, it’s not the guidelines itself, but it was that little paragraph in the article saying that we really don’t have the evidence and so that’s why it is what it is.”* Int #3  *“’Don’t examine your breast’ – it’s a weak recommendation”* Int #1 |
| **Changing a previous practice** |  | *“old habit, its routine when I examine the chest, some patients still want it done” (90-139)* | | *“It was awkward. I would say that in the first two to three weeks after we completed the module I personally tried quite hard to implement the guidelines, but it was very difficult because it was a break in the habit.”* Int #10 |
| **Time required to implement changes in practice** | *“I learned to bring up the topic only when I am NOT running behind schedule” (40-433)*  *Some pts want screening and a lengthy discussion can result” (80-348)* | *“Patient expectations/doubtfulness contributed to the time loss. There was resistance, disbelief and time spent empathetically listening to women’s testimonials about why they were more anxious to be unscreened” (40-433)*  *“time in the office for discussion patient’s own concerns about the need for mammograms if <50” (110-073)*  *“the time it takes to review pro’s / con’s with the patient” (80-350)* | | *“I’d have to say it was kind of awkward to keep it going because I was wasting a lot of time. My days were slow, I was getting annoyed with having to explain the new guidelines.”* Int#10 |
| **Fear of missing an abnormality or previous negative experience.** | *“I do struggle with not doing the exam. Recently before my practice change I saw a patient who had significant breast changes and dx with breast cancer who may never have been picked up on without the exam” (10-564)*  *“I’m still very anxious about missing a breast cancer diagnosis” ((30-105)* | *“my and the patient’s anxiety” (30-105)*  *“previous experience with breast cancer patients” (20-393)*  *“nervous about missing an abnormality” (80-345)*  *“have difficulty with not doing clinical exam as have picked up breast cancer” (20-390)* | | *“…one of the women [in PBSG group] had breast cancer in her 40s…so you can imagine how well that went…”* Int #1 |
| **Conflicting recommendations from other breast cancer screening programs** | *“current public perception mixed message by cancer care and NS breast screening program” (20-393)* | *“OBSP recalls patients” (80-341)*  *“Recommend screening mammogram in age 40-49 and perform CBE – because experts at BC Cancer Agency have expressed misgivings of new CTFPHC guidelines and my relationship with patients as individuals not statistics” (40-433)* | | *“I would say the breast cancer one is the only one that I have to say I don’t follow the guidelines, but that’s because our guidelines in BC are different.” Int #5*  *“There’s still the American Radiology Society, and I think the Canadian radiologists are still saying – I think I looked at this the other day – still saying mammograms in your 40s. Yeah, there’s still lots of conflicting. I have to look it up directly but I still think the radiology groups – both Canadian and American – are still recommending mammograms in your 40s. And a couple of European groups also.”* Int #3 |
| **Disagreement among PBSG group members as to interpretation of the evidence** | *“many of the group members did not agree with guidelines” (20-399)* | *“I was surprised to learn that the evidence is ‘weak’ for all issues which makes me think twice (e.g. for stopping clinical breast exam). The disagreement among my experienced colleagues makes it difficult to know what to do.” (20-390)* | | *“… when we have our meeting, you know, this one says, you know, ‘I tried it and this is what happened,’ and someone else said, “I just couldn’t bring myself to do this,” and someone else said, “Well, this is working okay, because my patients are good with it,” and so we... and that’s how we sort of separated us out according to who’s the ones with the older practices and the younger practices.”* Int #4 |
| **Patient expectations (e.g., to conduct a CBE/BSE) / concerns** | *“patient expectations. They don’t believe me. They think I’m being cheap & saving the government $ at the expense of their lives” (90-146)*  *“patients that I have been managing for a long time that are used to having breast exams done with their annual checkups” (80-344)* | *“patient expectations and my personal belief in CBE” (100-784)*  *“patients concerns or anxiety may lead to exams” (30-107)*  *“many of my patients will still want screening with mammos <50, scared to miss a breast cancer” (50-986)* | | *“… so the whole recommendation that clinical breast exam is no longer necessary, when people have been told for at least forty or fifty years…I’ve had a few people get really angry and tell me that we’re just cutting costs, we’re just ..trying to get them out faster”* Int #4  *…absolute benefit of screening with mammography, if you look at all those dots, it is an eye opener…but you know you can show this to patients, but their attitude is, …I don’t want that to be me…”* Int #14 |
| *PBSG=Practice Based Small Group; CBE=clinical breast exam; BSE=breast self-exam; pts=patients; US=United States; dx=diagnosis; BC=British Columbia (Canada); NS=Nova Scotia (Canada);; OBSP=Ontario Breast Screening Program* | | | | |
